# Comparative Phytochemical Investigation of the Sources of Ayurvedic Drug *Patha*: A Chromatographic Fingerprinting Analysis

**DOI:** 10.4103/0250-474X.62235

**Published:** 2010

**Authors:** K. K. Hullatti, M. S. Sharada

**Affiliations:** Department of Pharmacognosy, National College of Pharmacy, Shimoga-577 201, India; 1 DOS in Botany, University of Mysore, Mysore-570 006, India

**Keywords:** Bebeerine, *Cissampelos pareira*, *Cyclea peltata*, HPLC, HPTLC, *Stephania japonica*

## Abstract

Standardization of herbal drugs based on their chemical and biological activity profile is an important prerequisite for acquiring the herbal market. The main problem encountered in standardization of Ayurvedic drugs is proper identification of the source plant. The present study was aimed to establish identification characters, quality control parameters, chemical and biological parameters for roots of three plants *Cissampelos pareira, Cyclea peltata* and *Stephania japonica* (Fam. Menispermaceae) which are being used as source of *Patha*, in the market. All the three plant were subjected for evaluation of quality control parameters as per WHO guidelines and root extracts and total alkaloidal fractions were subjected for HPTLC and HPLC fingerprinting analysis using a marker compound Bebeerine isolated from roots of *Cissampelos pareira.* The parameters studied clearly indicated the significant differences among the three plant materials. The roots of *Cissampelos pareira* can be distinguished from other two plants by presence of high concentration of alkaloids especially the presence of high concentration of pharmacologically active alkaloid bebeerine, which was found to be present in very low concentration in *Stephania japonica* and absent in roots of *Cyclea peltata*. The roots of *Cyclea peltata* were found to contain high concentration of saponins and comparatively in low concentration in *Cissampelos pareira* where as it was found to be absent in roots of *Stephania japonica*.

Numbers of scientific documentations are available on crude drug extracts, promoting these herbal drugs in international/national market is difficult due to lack of reproducible biological reports, Selection of wrong plants and lack of data on the time and area of collection and Identity of the botanical source.

In certain cases confusion exists in the identity of the source material where the origin of particular drug is assigned to more than one plants, sometimes having different morphological and taxonomical characters. The Ayurvedic drug *Patha* is one such drug. The roots of *Cissampelos pareira* L., ver. *hirsuta* and *Cyclea peltata* (Lam.) J. Hooker and Thoms. (Family-Menispermaceae) are being used as *Patha* in Ayurvedic system of medicine. The roots of *Cissampelos pareira* is known as *LaghuPatha* and *Cyclea peltata* as *RajaPatha* in various literatures, but *Charaka* samhita, *Sushruta* and *Vagbhat* have not mentioned two types of *Patha*[1]. Another plant *Stephania japonica* (Thunb.) Miers. (Family-Menispermaceae) is being used as substitute for *Patha*[2]. According to Kirtikar and Basu[3], *Stephania japonica* (Syn. *Stephania hernandifolia)* is equated as *Patha* and *Cissampelos pareira* as *LaghuPatha*.

The Ayurvedic Pharmacopoeia of India recognizes roots of *Cissampelos pareira* as *Patha*, but in the same the synonym in Ayurveda is mentioned as *Ambusthaki* in Sanskrit and *Patha* in Malayalam. According to Prajapathi in Agro's dictionary of medicinal plants, roots of *Cissampelos pareira* is mentioned as *Ambustha, Cyclea peltata* as *Patha* and *Stephania japonica* as *Vanatiktaka*[4].

*Patha* is useful in colicky pains, fever, vomiting, skin conditions, cardiac pains, burning feeling, pruritus, poisons, breathing difficulties and worms[5]. The therapeutic uses of *Patha* according to the Ayurvedic Pharmacopoeia of India are in the treatment of abdominal pain, diarrhoea, skin diseases, pruritus and fever[6].

With this background, in the present study an attempt was made to systematically evaluate Ayurvedic drug *Patha*. The plants selected for the study include *Cissampelos pareira, Cyclea peltata* and *Stephania japonica.* The study was aimed to establish quality control parameters and to investigate the phytochemical parameters including HPTLC and HPLC fingerprinting profile.

## MATERIALS AND METHODS

### Quality control parameters:

All the Quality Control Parameters, such as Solvent extractive values, ash values, crude fibre content, foaming index, tannin content and swelling index were determined as prescribed by the WHO, to incorporate any natural drug into the herbal pharmacopoeia[7]. Total phenol content of the extracts was determined by using the Folin-Ciocalteu method[8]. The amount of total alkaloids present in the three plants was determined by modification of method given by Rajpal[9].

### Phytochemical evaluation:

The coarsely powdered plant material was subjected to successive extraction in a Soxhlet apparatus using petroleum ether, chloroform and methanol. The extraction was performed at 55°, for 8 to 10 h (till the coloured extract became colourless). The extracts were filtered while hot and subjected to concentration under vacuum using a rotary flash evaporator. The percentage yield of the extract was calculated based on the weight of air dried plant material.

The different qualitative chemical tests were performed for establishing the chemical profiles of the prepared extracts. The petroleum ether, chloroform and methanolic extracts of three plants were subjected to the preliminary chemical tests for the identification of various phytoconstituents[10].

### Isolation of total alkaloids:

One hundred grams of the methanol extract was dissolved in dil. H_2_ SO_4_ (5% v/v). Solution was filtered and the pH of the filtrate was adjusted to 9.5 with dilute ammonia and the free alkaloids were extracted with chloroform and the process was repeated thrice. Finally, the chloroform layer was concentrated to dryness to get the total alkaloidal fraction. Marker compound was isolated in pure form by following the method by Kupchan *et al*.[11] with modifications.

### High performance thin layer chromatography:

High performance thin layer chromatography was performed with Camag HPTLC system with Linomat IV sample applicator device, twin trough development chamber No. 022-5155, HPTLC plate- silica gel GF_254_, Camag cats TLC plate scanner with Camag integration software.

Crude extracts was prepared by dissolving 10 mg of the extract in 10 ml of methanol by warming and intermittent shaking. For total alkaloids 10 mg of the sample was dissolved in 10 ml of chloroform by intermittent shaking. Solvent system used was *n*-butanol:ethyl acetate:formic acid: water (30:50:10:10) for crude extracts and cyclohexane:chloroform:diethylamine (50:40:10) for total alkaloids. The plates were scanned at 365 nm (for crude extracts) and at 295 nm (for total alkaloids).

### High-performance liquid chromatography:

HPLC analysis was performed on a Shimadzu LC-10AD VP system equipped with a binary gradient system and SPD-M10A VP photodiode array (PDA) detector. A Hypersil Gold HPLC column (100 mm×4.6 mm, 3 μm) was used for all experiments. 50 μl of the sample was injected into SIL-10AD VP auto sampler. The initial chromatographic conditions were 100% water containing 10 mM ammonium acetate and the pH was adjusted to 9.5 with ammonia (25% v/v). After injection, the chromatographic conditions were gradually changed through a linear gradient profile to 90% acetonitrile and 10% of the original aqueous mobile phase in 50 min. These conditions were kept stable for 5 min thereafter the column was re-equilibrated to the initial conditions. The flow rate was kept constant at 1 ml/min. The samples for HPLC were prepared by dissolving 3 mg of the dried material in 3 ml of acetonitrile and filtering through a 0.45 μm (Nylon) filter into HPLC vials.

## RESULTS AND DISCUSSION

All the quality control parameters studied clearly indicate the significant differences among the studied three plants. The marpho-anatomical studies also have shown the various distinguishing characters among these three plants[12]. Especially absence of saponins in roots of *Stephania japonica* becomes the important tool to distinguish it from other two plants. High saponin content, low water soluble ash and low polyphenol content of *Cyclea peltata* is another important differentiating factor (Table 1). Over all these parameters play an important role in identification and quality control of these three medicinal plants.

**TABLE 1:** PHYSICO-CHEMICAL CONSTANTS OF THE PLANT MATERIALS

Parameters (% w/w)	*Cissampelos pareira*	*Cyclea peltata*	*Stephania japonica*
Extractive value			
Ether soluble	4.95±0.44	9.04±0.51	4.00±0.74
Alcohol soluble	11.5±0.46	15.8±1.18	9.09±1.48
Water soluble	12.93±1.72	17.98±0.58	12.56±2.90
Ash value			
Total ash	3.72±0.09	6.18±0.12	6.10±0.20
Water soluble ash	0.87±0.09	0.06±0.01	1.93±0.13
Acid insoluble ash	0.54±0.05	1.09±0.25	0.41±0.08
Crude fibre	22.23±0.28	17.48±0.12	27.34±0.21
Foaming index	169.52±23.46	350.00±80.07	< 100.00
Swelling index	4.22±0.16	3.56±0.11	4.03±0.13
Total poly phenols (mg/g as gallic acid eq.)	347.58±11.85	191.08±3.7	356.44±23.18
Total tannins	14.78±0.93	8.06±0.53	10.80±0.62
Total bitters	0.52±0.06	2.22±0.07	1.56±0.06
Total alkaloids	6.61±0.42	5.50±0.43	4.17±0.83

Values are mean of 10 readings ±SEM

**Fig. 1:**
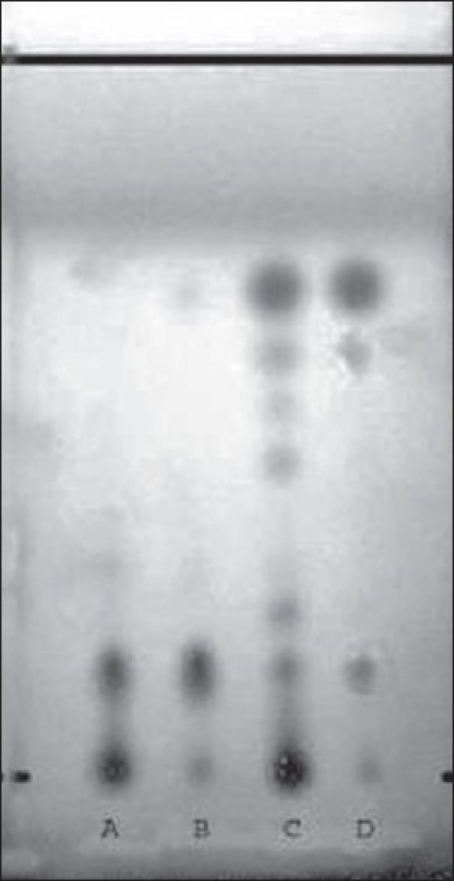
TLC profile of *Cissampelos pareira* and *Cyclea peltata* root extract Mobile phase-cyclohexane:chloroform:diethylamine (40:50:10), where track A is *Cissampelos pareira* root extract, track B is fraction A of *Cissampelos pareira* root extract, track C is *Cyclea peltata* root extract and track D is fraction A of *Cyclea peltata* root extract

The alkaloids isolated from alcoholic root extracts of *Cissampelos pareira* and *Cyclea peltata* were subjected for a detailed evaluation. The chromatographic evaluation indicated that the isolated compound from *Cissampelos pareira* was pure as a single spot was obtained in TLC evaluation whereas the compound from *Cyclea peltata* was found to be mixture of three alkaloids (fig. 1). Compound exhibited positive response for Dragendorff's reagent indicating that the isolated compound was alkaloid. The melting point of compound was found to be 157-158°. The λ_max_ of the compound was determined in chloroform, methanol and 2.5% hydrochloric acid using a Shimadzu 160-A UV/Vis spectrophotometer. The compound has showed almost same absorption maxima in the different solvents *viz.* chloroform (282 nm), methanol (281.5 nm) and 2.5% hydrochloric acid (281 nm).

**Fig. 2:**
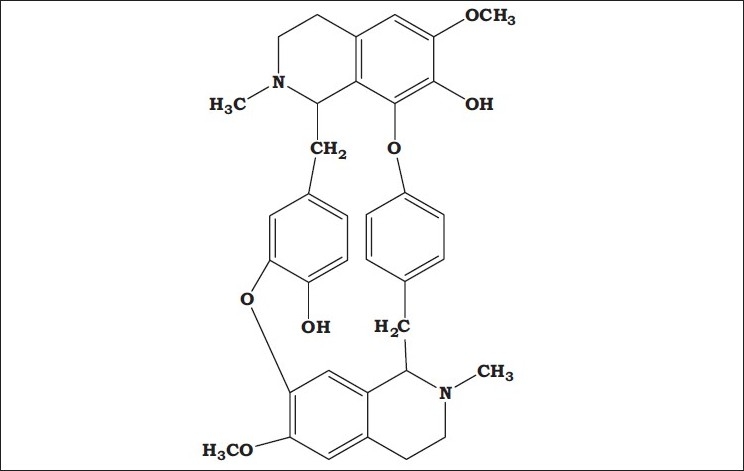
− (−) bebeerine

The FT-IR spectrum of Fraction A of *Cissampelos pareira* showed the presence of phenolic O-H stretching at 3434 cm^−1^, C-H stretching of CH_3_ at 2941, C-H stretching of CH_2_ at 2842, aromatic C^3^=C ring stretch at 1507, 1463, 1452 cm^−1^, C-N stretch at 1330 cm^−1^, C-O stretch at 1232, ether (C-O-C) stretch at 1122 cm^−1^ and C-H bend at 834 cm^−1^, C=C bend at 797 and O-H bend at 584 cm^−1^. The similar bands have been observed in the FT-IR spectrum of the standard −(−) Bebeerine. The ^1^H-NMR spectrum of the fraction displayed four doublet signals for aromatic C-H at δ~7.2 to 6.6, singlet at δ~6.5 and 6.0 for O-H, a triplet at δ~3.8 for methoxy of O-CH_3_, doublet at δ~2.8, 2.7 and 2.6 for methylene of -CH_2_ - and N-CH_2_-(heterocyclic ring) and a triplet at δ~2.3 for methyl of N-CH3. Similar peaks were observed in the ^1^ H-NMR spectrum of standard −(−) bebeerine (fig. 2).

**Fig. 3:**
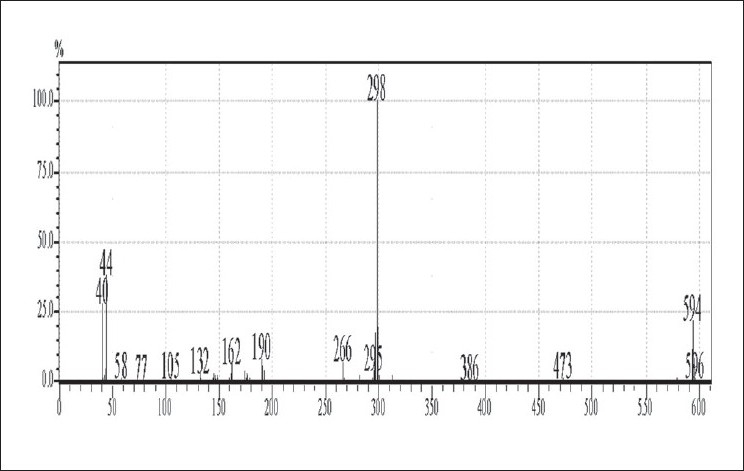
Mass spectra of *Cissampelos pareira* alkaloidal fraction

**Fig. 4:**
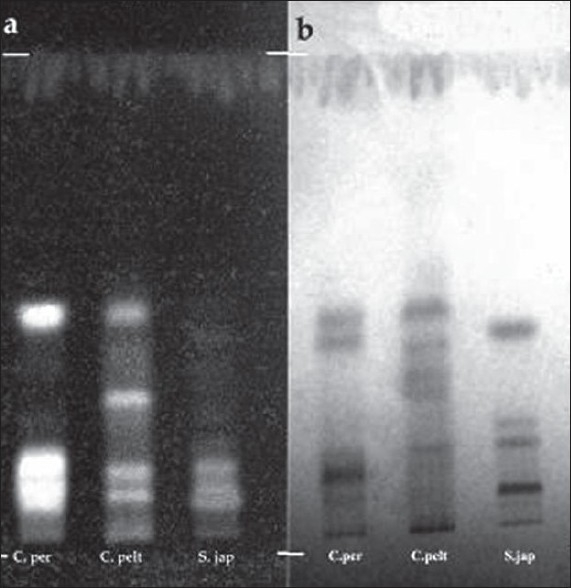
HPTLC profile of methanol extracts. Mobile phase is n-butanol:ethyl acetate:formic acid:water (30:50:10:10), where a. chromatogram visualized under UV 254 nm, b. chromatogram visualized under UV 365 nm, C. per is *Cissampelos pareira*, C. pelt is *Cyclea peltata* and S. Jap is *Stephania japonica*

**Fig. 5:**
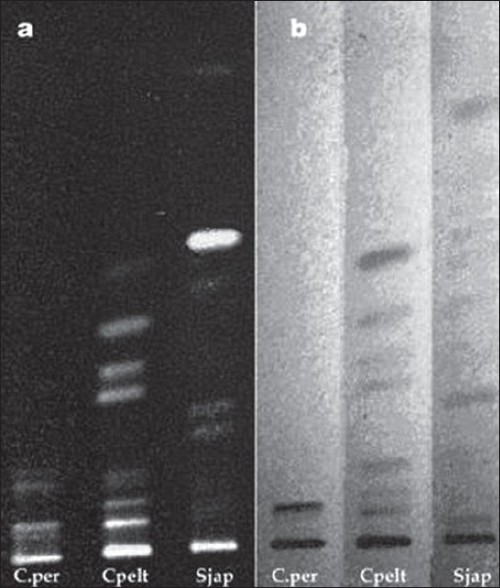
HPTLC profile of total alkaloids Mobile phase is cyclohexane:chloroform:diethylamine (40:50:10), where a. chromatogram visualized under UV 254 nm, b. chromatogram visualized under UV 365 nm, C. Per is *Cissampelos pareira*, C. pelt is *Cyclea peltata* and S. jap is *Stephania japonica*

**Fig. 6:**
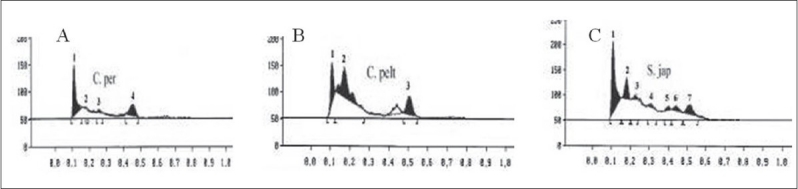
HPTLC chromatogram of root extracts A. *Cissampelos pareira* B. *Cyclea peltata* C. *Stephania japonica*

**Fig. 7:**
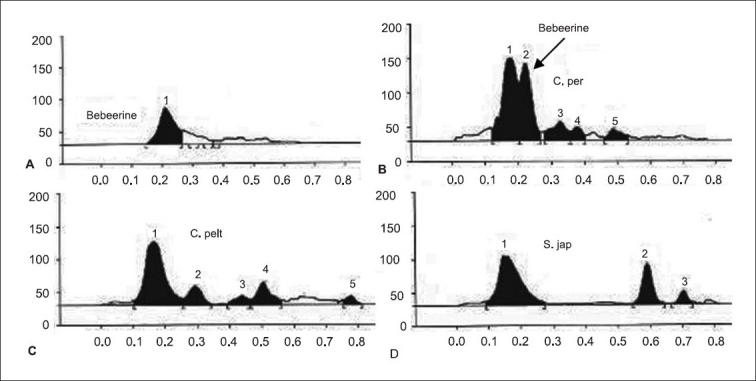
HPTLC chromatogram of total alkaloids A. [−(−) Bebeerine] B. *Cissampelos pareira C. Cyclea peltata D. Stephania japonica*

The GC-MS studies showed that the retention time of the fraction was found to be 20.07 min. Molecular weight of the compound has been confirmed by Mass spectroscopy (fig. 3). In the HPTLC studies, the same solvent systems as used in TLC were used for the separation. The number of compounds separated, their R_f_ values and their relative percentage area were noted (figs. 4 and 5). These results are shown in figs. 6 and 7. The methanol extract of *Cissampelos pareira* showed the presence of 4 peaks and 2 of them with R_f_ values 0.11 and 0.45, were found to be the major ones with about 76 and 17% peak areas, respectively. Similarly the extracts of *Cyclea peltata,* and *Stephania japonica* exhibited 6 and 7 spots, respectively. These extracts were found to contain 3 (47, 31 and 21%), and 3 (62, 16, and 7%) major spots, respectively. The peak at R_f_ value 0.11 was found to be common in all the three plants.

The total alkaloid fraction was also subjected for HPTLC studies using a different solvent system. The solvent system used was cyclohexane:chloroform:diethylamine (50:40:10). Bebeerine isolated from *Cissampelos pareira* was used as the internal standard. The number of compounds separated and their respective R_f_ are noted. The total alkaloids of *Cissampelos pareira* showed 5 peaks of which two are major peaks at R_f_ of 0.18 and 0.23. The peak with the R_f_ of 0.23 corresponds to that of bebeerine. The total alkaloids of *Cyclea peltata* showed 5 peaks of which three are major with R_f_ values of 0.17, 0.30 and 0.51, but none of them matched with marker compound bebeerine. The total alkaloids of *Stephania japonica* showed 3 peaks at an R_f_ of 0.16, 0.59 and 0.71.

**Fig. 8:**
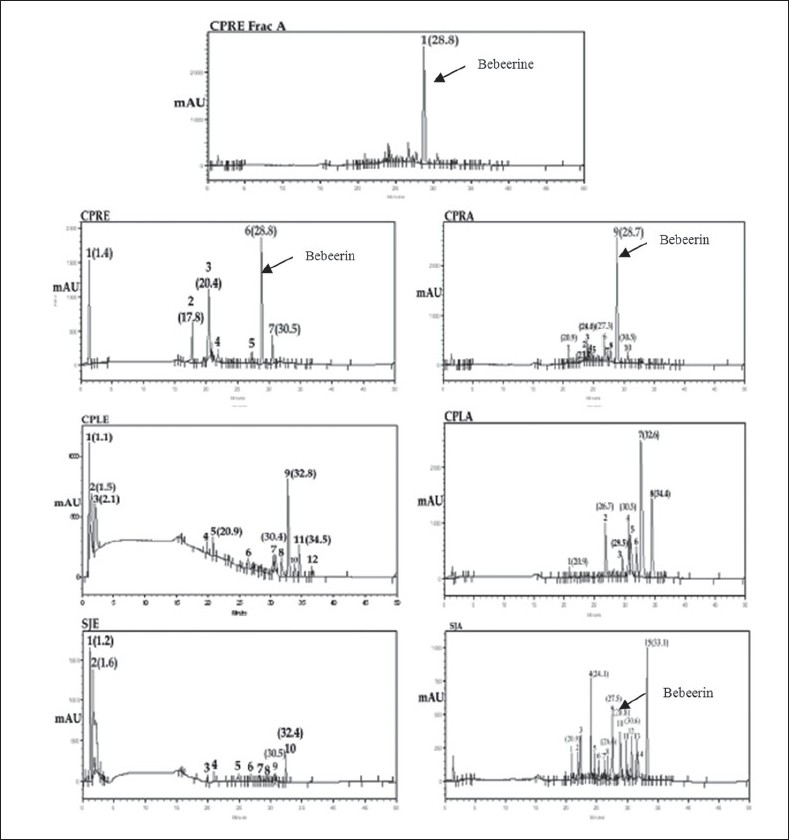
HPLC Chromatograph of methanol root extracts and total alkaloidal fraction CPRE is *Cissampelos pareira* root extract, CPRA *is Cissampelos pareira* total alkaloids, CPLE is *Cyclea peltata* root extract, CPLA is *Cyclea peltata* total alkaloids, SJE is *Stephania japonica* root extract and SJA is *Stephania japonica* total alkaloids

The three plant materials obtained from different genus of Menispermaceae family were analyzed to evaluate the distribution of bio-constituents. After fingerprint analysis by HPLC method, the qualitative results of the peak groups were significantly different in all the three plants. However they are similar in certain constituents but once again significantly different on quantitative basis. In particular with the marker compound −(−) bebeerine, which was found to be the principle constituent of roots of *Cissampelos pareira* (peak −6 with R_t_ of 28.8 and ratio of peak area of 28.9 in methanol extract and peak −9 with R_t_ 28.7 with ratio peak area of 53.9 in total alkaloids). This constituent was found to be absent in roots of *Cyclea peltata* where as it is found to be present in small quantity in roots of *Stephania japonica* (Undetected in extract and peak −10 with R_t_ 28.8 and ratio peak area of 5.1 in total alkaloids). Similarly two components were found to be common in methanol extract of all the three plants. Peak −1 and 7 (*Cissampelos pareira)*, peak −2 and 7 (*Cyclea peltata*) and peak −2 and 9 (*Stephania japonica*). Once again they differ significantly on quantitative basis. Three components were found to be common in total alkaloidal fraction of these three plants. Peak – 1(R_t_ −20.9), 6 (R_t_ −26.7) and 10 (R_t_ −30.5) in *Cissampelos pareira.* Peak – 1(R_t_ −20.9), 2 (R_t_ −26.7) and 4 (R_t_ −30.5) in *Cyclea peltata*. Peak – 1(R_t_ −20.9), 8 (R_t_ −26.6) and 12 (R_t_ −30.6) in *Stephania japonica*. These constituents more or less differ quantitatively in each plant (fig. 8).

It was also observed that the studied pharmacological activities were also significantly different in all the three plants. The methanol root extract of *Cissampelos pareira* has shown the significant antipyretic activity where as, other two plants failed to produce significant antipyresis in the animal models[13]. All the three plants have exhibited varying degree of antimicrobial activity[14]. All these studied parameters clearly indicate that the roots of *Cissampelos pareira* can be used as the source of *Patha* as it fulfils the Ayurvedic claims of this drug. Still the further detailed studies with respect to the other pharmacological actions of these three plants need to be compared.
